# Axonal stimulation affects the linear summation of single-point perception in three Argus II users

**DOI:** 10.1101/2023.07.21.23292908

**Published:** 2023-07-26

**Authors:** Yuchen Hou, Devyani Nanduri, Jacob Granley, James D. Weiland, Michael Beyeler

**Affiliations:** 1 Department of Computer Science, University of California, Santa Barbara, CA; 2 Department of Psychological & Brain Sciences, University of California, Santa Barbara, CA; 3 Department of Biomedical Engineering, University of Southern California, Los Angeles, CA; 4 Department of Biomedical Engineering, University of Michigan, Ann Arbor, MI

**Keywords:** Retinal prosthesis, phosphene shape, pattern vision

## Abstract

**Purpose.:**

Retinal implants use electrical stimulation to elicit flashes of light (“phosphenes”). Single-electrode phosphene shape has been shown to vary systematically with stimulus amplitude and frequency as well as the retinal location of the stimulating electrode, due to incidental activation of passing nerve fiber bundles. However, this knowledge has yet to be extended to paired-electrode stimulation.

**Methods.:**

We retrospectively analyzed 4402 phosphene drawings made by three blind subjects implanted with an Argus II Retinal Prosthesis. Phosphene shape (characterized by area, perimeter, major and minor axis length; normalized per subject) and number of perceived phosphenes were averaged across trials and correlated with the corresponding single-electrode parameters. In addition, the number of phosphenes was correlated with stimulus amplitude and neuroanatomical parameters: electrode-retina (“height”) and electrode-fovea distance (“eccentricity”) as well as the electrode-electrode distance to (“between-axon”) and along axon bundles (“along-axon”). Statistical analyses were conducted using linear regression and partial correlation analysis.

**Results.:**

Simple regression revealed that each paired-electrode shape descriptor could be predicted by the sum of the two corresponding single-electrode shape descriptors (p<.001). Multiple regression revealed that pairedelectrode phosphene shape was primarily predicted by stimulus amplitude, electroderetina distance, and electrode-fovea distance (p<.05). Interestingly, the number of elicited phosphenes increased with between-axon distance (β=.162,p<.05), but not with along-axon distance (p>.05).

**Conclusions.:**

The shape of phosphenes elicited by paired-electrode stimulation was well predicted by the shape of their corresponding single-electrode phosphenes, suggesting that two-point perception can be expressed as the linear summation of single-point perception. We also found that the number of perceived phosphenes increased with the between-axon distance of the two electrodes, providing further evidence in support of the axon map model for epiretinal stimulation. These findings contribute to the growing literature on phosphene perception and have important implications for the design of future retinal prostheses.

## 1. Introduction

Retinitis pigmentosa (RP) is an inherited degenerative disease of the eye that is estimated to affect one in 4,000 individuals worldwide (Hamel, 2006). Although recent advances in gene and stem cell therapies (e.g., Russell et al., 2017, da Cruz et al., 2018; for a recent review see McGregor, 2019) as well as retinal sheet transplants (e.g., Foik et al., 2018, Gasparini et al., 2019; for a recent commentary see Beyeler, 2019) are showing great promise as near-future treatments for early-stage RP, electronic retinal prostheses continue to be a pertinent option for later stages of the disease.

Retinal prostheses typically acquire visual input via an external camera, which is then translated into electrical pulses sent to a microstimulator implanted in the eye (Weiland et al., 2016). The stimulator receives the information, decodes it, and stimulates the surviving retinal neurons with electrical current, thus evoking the perception of flashes of light (“phosphenes”). The most widely adopted retinal implant thus far is the Argus II Retinal Prosthesis System (Vivani Medical, Inc; formerly Second Sight Medical Products, Inc.), which was the first retinal implant to obtain regulatory approval in the US and Europe, and has been implanted in roughly 500 patients worldwide (Luo and da Cruz, 2016).

A series of papers demonstrated that phosphenes elicited by stimulating a single Argus II electrode have a distinctive shape that is relatively consistent over time (Nanduri et al., 2008; Luo et al., 2016; Beyeler et al., 2019). Phosphene shape has been shown to depend strongly on the retinal location of the stimulating electrode, predominantly elongated along the trajectory of the underlying nerve fiber bundle (Rizzo et al., 2003; Beyeler et al., 2019). In addition, phosphene appearance varies systematically with stimulus amplitude and frequency (Horsager et al., 2009; Nanduri et al., 2012; Sinclair et al., 2016) to the extent that a simple computational model can predict phosphene shape across a wide range of stimulus parameters (Granley and Beyeler, 2021).

However, less is known about how phosphenes combine when multiple electrodes are stimulated. Early research suggested that repeated paired stimulation resulted in reproducible phosphenes as subjects perceived “similar” phosphenes on 66% of trials (Rizzo et al., 2003). But more recent studies indicated that phosphenes tend to merge in nontrivial ways. For instance, Wilke et al. (2011b) highlighted the importance of electric crosstalk between electrodes in determining the response to simultaneous stimulation of multiple electrodes. Horsager et al. (2011) found that elicited percepts were affected by other stimulating electrodes (even after temporally staggering pulses to remove electric field interactions) and demonstrated a linear combination of threshold currents for simultaneous stimulation. Using a suprachoroidal prosthesis, Sinclair et al. (2016) found that bipolar electrode configurations produced percepts that were similar in appearance to the summation of the phosphenes that were elicited from the two individual electrodes using a monopolar configuration. Most recently, Yücel et al. (2022) identified several factors that might limit the spatial resolution of prosthetic vision, which included retinal damage, electrode-retina distance, and the inadvertent stimulation of nerve fiber bundles. To avoid electric crosstalk and aid the perceptual merging of multi-electrode phosphenes, some researchers (Beauchamp et al., 2020; Oswalt et al., 2021; Christie et al., 2022) considered sequential stimulation paradigms. However, sequential stimulation does not always lead to perceptually intelligible forms or objects; often subjects are only able to trace an outline of the perceived shape, and their interpretation of the shape relies heavily on this basic outline (Christie et al., 2022). Therefore, understanding how multi-electrode stimulation can be leveraged to produce form vision remains an open challenge for the field of visual prosthetics.

Here we aim to study the consistency and predictability of the (presumably) fundamental building blocks of form vision: the percepts elicited by single- and paired-electrode stimulation. While single-electrode stimulation is relatively well understood (Nanduri et al., 2008; Sinclair et al., 2016; Luo et al., 2016; Beyeler et al., 2019; Granley and Beyeler, 2021), it remains to be demonstrated whether this knowledge can be extended to predict phosphene appearance elicited by paired-electrode stimulation. Specifically, the axon map model (Beyeler et al., 2019; Granley and Beyeler, 2021) predicts that the probability of seeing two phosphenes should increase with increasing distance between their axon bundles (as opposed to distance on the retinal surface). To assess whether phosphenes sum linearly, and to determine which neuroanatomical and stimulus parameters may be predictive of paired-phosphene appearance, we retrospectively analyzed an extensive psychophysical dataset collected with the help of three Argus II patients.

## 2. Methods

### 2.1. Participants

This study involved three blind participants (one female and two male) with severe RP, ranging from 41 to 70 years in age at implantation (Table 1). Subjects were chronically implanted with the Argus II Retinal Prosthesis System as part of an interventional feasibility trial (clinicaltrials.gov NCT00407602; completed). All psychophysical experiments were carried out at least six months after device implementation. The study was approved by the Institutional Review Board (IRB) at each subject’s clinical site and was conducted under the tenets of the Declaration of Helsinki. Informed consent was obtained from the participants after explanation of the nature and possible consequences of the study.

Due to their geographic location, the participants were not directly examined by the authors of this study. Instead, initial experimental procedures were sent to the clinical site, and trained field clinical engineers performed the experiments as specified. Raw collected data was then sent to the authors for subsequent analysis.

### 2.2. Stimuli

Argus II consists of a 6 × 10 grid of platinum disc electrodes, each 200 μm in diameter, subtending 0.7° of visual angle (Luo and da Cruz, 2016). Electrodes were spaced 575 μm apart. In day-to-day use, an external component is worn by the user, consisting of a small camera and transmitter mounted on a pair of glasses. The camera captures video and sends the information to the visual processing unit (VPU), which converts it into pulse trains using pre-specific image processing techniques (*camera mode*).

All stimuli described in this study were presented in *direct stimulation* mode. Stimuli were charge-balanced, cathodic-first, square-wave pulse trains with 0.45 ms phase duration and 250 ms total stimulus duration. Stimulus amplitudes, frequencies, and the number of stimulated electrodes varied based on the design of each experiment. Stimuli were programmed in Matlab using custom software, and pulse train parameters (i.e., the electrode(s) to be stimulated, current amplitude, pulse width, inter-pulse interval, and overall stimulus duration) were sent directly to the VPU, which then sent the stimulus commands to the internal portion of the implant using an inductive coil link. The implanted receiver wirelessly received these data and sent the signals to the electrode array via a small cable.

### 2.3. Psychophysical methods

Perceptual thresholds for individual electrodes were measured using an adaptive yes/no procedure implemented using custom software (see Appendix A).

Participants were asked to perform a drawing task upon electrical stimulation of the retina. Subjects were comfortably seated in front of a touchscreen monitor whose center was horizontally aligned with the subject’s head. The distance between the subject’s eyes and the monitor was 83.8 cm for Subject 1, 76.2 cm for Subject 2, and 77.5 cm for Subject 3.

Each stimulus was presented in 5–10 trials randomly amongst other stimuli with different frequency and/or amplitude levels. The stimulus frequency ranged from 6 Hz to 120 Hz, and the amplitude was between 1.25 times threshold to 7.5 times threshold. Within each trial, either one or two electrodes were randomly selected and stimulated; if two electrodes were selected, they were stimulated simultaneously. After delivering each stimulus and before moving to the subsequent trial, subjects were asked to trace the perceived shape on the touchscreen monitor. Shapes were closed by automatically connecting the first and last tracked fingertip location, after which a flood-fill was applied. The drawing data was recorded and converted into a binary shape data file using Matlab, and stored for future analysis. All psychophysical experiments were carried out by local field clinical engineers at each participating site, and the results were forwarded to the authors.

Since the validity and reliability of the experiment relied on the ability of our subjects to accurately draw the perceived phosphenes, a control task was conducted where subjects were asked to feel six different tactile shapes made of felt with a cardboard background, and then draw them on a touchscreen (Beyeler et al., 2019). As the shape of these tactile targets was known and we asked subjects to repeat each drawing five times, we were able to determine each subject’s drawing error and bias. A detailed description of this task can be found in the Appendix S2 of Beyeler et al. (2019). In short, this control established baseline drawing variability for each subject, against which we could compare electrically elicited phosphene drawing variability to determine the stability of phosphene appearance.

### 2.4. Phosphene shape descriptors

**Figure 1:**
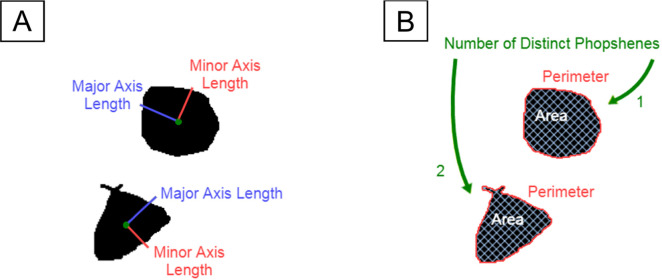
An example of a phosphene drawing and five shape properties of the phosphene. A) Phosphene described by major axis length (red) and minor axis length (blue). B) Phosphene described by area (white), perimeter (red), and the number of distinct regions (green).

Phosphene shape was quantified using four parameter-free shape descriptors commonly used in image processing: area, perimeter, major axis length, and minor axis length (Nanduri et al., 2008). An example is shown in Fig. 1. These descriptors are based on a set of statistical quantities known as *image moments*. For an M×N pixel grayscale image, I(x,y), where x∈[1,M] and y∈[1,N], the raw image moments $M_{ij}$ were calculated as:

(1) $$M_{ij}=\sum_{x}\sum_{y}x^{i}y^{j}I\left(x,y\right).$$

Raw image moments were used to compute area $\left(A=M_{00}\right)$ and the center of mass $(\overset{‾}{x},\overset{‾}{y})=\left(M_{10}/M_{00},\;M_{01}/M_{00}\right)$ of each phosphene.

Phosphene major/minor axis lengths were calculated from the covariance matrix of the phosphene drawing:

(2) $$cov[I(x,\;y)]=[\begin{array}{ll} \mu_{20}^{\prime } & \mu_{11}^{\prime }\\ \mu_{11}^{\prime } & \mu_{02}^{\prime } \end{array}],$$

where $mu_{20}^{\prime }=M_{20}/M_{00}-{\overset{‾}{x}}^{2},\;\mu_{11}^{\prime }=M_{11}/M_{00}-\overset{‾}{x}\overset{‾}{y}$, and $\mu_{02}^{\prime }=M_{02}/M_{00}-{\overset{‾}{y}}^{2}$. The eigenvectors of this matrix corresponded to the major and minor axes of the image intensity.

Phosphene perimeter was calculated using an algorithm described in Benkrid et al. (2000), which approximates the length of each phosphene’s contour as a line running through the centers of connected border pixels.

Phosphene orientation was previously shown to depend mostly on the retinal location of the stimulating electrode (Beyeler et al., 2019) and was thus excluded from the main analysis. However, the interested reader is referred to Appendix E for the supplemental analysis.

If a drawing had more than one phosphene, each shape descriptor was extracted for each perceived phosphene, summed over all phosphenes within a drawing (to account for a variable number of elicited phosphenes), and then averaged across trials.

### 2.5. Estimation of electrode-fovea distance and inter-electrode distance

**Figure 2:**
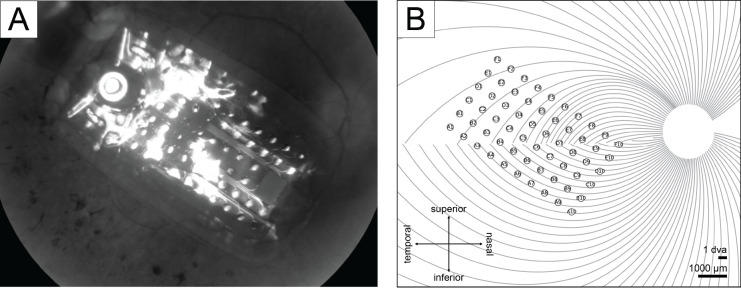
A) Subject 2’s fundus image with Argus II implant placed over the retinal surface. B) Subject 2’s simulated implant placed on the simulated axonal map.

Electrode-fovea distances and inter-electrode distances were estimated using the *pulse2percept* software (Beyeler et al., 2017). Following Beyeler et al. (2019), each subject’s implant location was estimated based on the fundus images taken before and after surgery by extracting and analyzing retinal landmarks (e.g., foveal region and optic disc). Image pixels were converted into retinal distances using Argus II inter-electrode spacing information. The implant image was then rotated and transformed such that the raphe fell on the horizontal axis and the fovea was the origin of the new coordination system. The stimulated implant was placed on a simulated map of axonal nerve fiber bundles (Fig. 2), which was modeled based on 55 healthy subjects’ ophthalmic fundus photographs (Jansonius et al., 2009). Since the fovea is the origin in the stimulated implant’s coordinates, the electrode-fovea distance was measured as the distance between an electrode and the origin.

**Figure 3:**
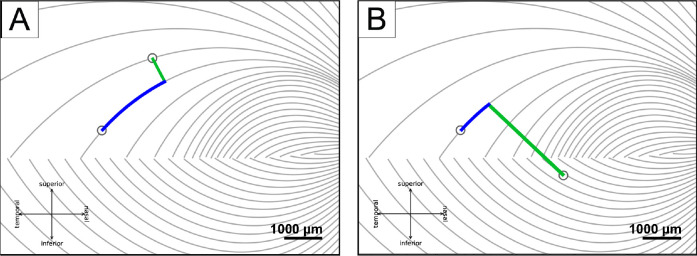
Axonal distances (adapted from Yücel et al., 2022). A) The between-axon distance (green line) and the along-axon distance (blue curve) when two electrodes are on the same side of the raphe, B) and when two electrodes are on different sides.

Inter-electrode distance measurements were adapted from Yücel et al. (2022) to investigate the effect of axonal stimulation on perceived phosphene shapes, in which the distance between two electrodes was divided into two, nearly orthogonal components:

- *between-axon* distance (green lines in Fig. 3): the shortest distance between the center of the more nasal electrode to the closest axon of the more temporal electrode;
- *along-axon* distance (blue curves in Fig. 3): the distance from the center of the temporal electrode, along the nasal electrode’s closest axon, up to the point where the nasal electrode’s between-axon line reached the temporal electrode’s axon.

### 2.6. Estimation of electrode-retina distance

Electrode-retina distances were estimated from post-surgical OCT images collected with either Cirrus HD-OCT (Carl Zeiss Inc) or Topcon 3D-OCT 1000 (Topcon Inc). The SD-OCT scans were obtained 6 months after Subjects 1’s and 2’s implantation and 13 months after Subject 3’s implantation.

**Figure 4:**
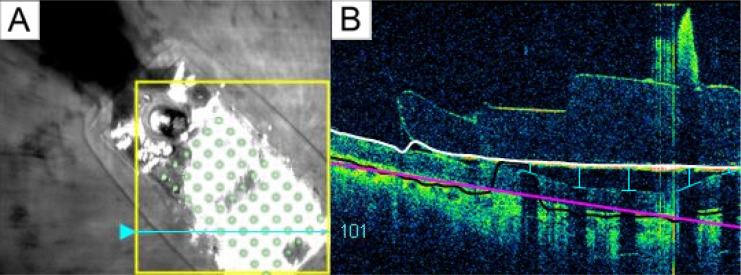
A) Subject 1’s retinal implant fundus image. The cyan arrow marked the current scanning area, and the green electrode array was superimposed onto the original image for better electrode visualization. B) Subject 1’s OCT b-scan. Each electrode-retina distance (vertical blue line) was represented by the length between the center of the shadow on the retinal surface (horizontal blue line) and the implant (white line).

When performing OCT scanning, the opaque metal electrodes prevent image acquisition directly underneath the corresponding electrode. However, based on the length of the shadow between the electrode and the retinal surface, it is possible to estimate the electrode-retina distance of that electrode (Ahuja et al., 2013). A single grader manually measured the electrode-retina distance by counting the number of pixels from the center of the shadow on the retinal pigment epithelium to the implant (Fig. 4) and converting the pixel distances to microns. Distances of poorly imaged electrodes were excluded from the dataset.

Details about each subject’s estimated electrode-fovea distances and electrode-retina distances are given in Table 2. Welch’s t-test was used to compare subjects’ differences in stimulus and neuroanatomical parameters. There was no statistical difference between the averaged electrode-fovea distance across different subjects (for Subjects 1 and 2: t(29)=1.529, p>.05; for Subjects 2 and 3: t(29)=0.114,p>.05; for Subjects 1 and 3: t(29)=−1.247,p>.05). In terms of electrode-retina distance, Subject 1 had significantly larger values than the other two subjects (t(29)=5.776,p<.001 and t(29)=5.776,p<.001) whose implant was closely attached to the retina.

### 2.7. Data cleaning

To make the collected phosphene drawings amenable to automated image analysis, we manually inspected all 4402 drawings to make sure that:

- all drawn contour lines were closed (e.g., when drawing a circle, the starting point of the drawing must touch the endpoint);
- small specs and other artifacts (most likely caused by accidentally touching the touchscreen) were not counted as additional phosphenes.

This procedure is explained in detail in Appendix B. Less than 1% of the 4402 phosphene drawings were identified as needing to be cleaned, owing to the precision with which the original data was collected.

### 2.8. Statistical analysis

Data entry and statistical analyses were performed in Python (version 3.8.12, Python Software Foundation). Python package scikit-image (version 0.18.3, https://scikit-image.org) was used for calculating different phosphene shape properties, and matplotlib (version 3.5.0, https://matplotlib.org) was used for presenting phosphene drawings and analysis plots.

To facilitate the regression analyses as well as to control for individual drawing bias and variance (Beyeler et al., 2019), we standardized the data as follows. First, the dependent variables, which describe phosphene shape (i.e., area, perimeter, major/minor axis lengths) were expressed as multiples of the shape descriptors elicited by a “standard” pulse train (amplitude: 2× threshold, frequency: 20 Hz). This procedure was performed separately for each subject, but considered drawings from all recorded electrodes of that subject, in order to account for drawing bias and variance. For instance, the area of an individual phosphene was normalized by the phosphene area averaged across all drawings of a particular subject when one of their electrodes was stimulated with the standard pulse train. Second, shape descriptors were first extracted from each individual drawing, and then averaged across trials of the same electrode and stimulus combination, in order to eliminate repeated measures of the same data point. Third, all independent variables (i.e., amplitude, frequency, electrode-retina distance, electrode-fovea distance, between-axon distance, and along-axon distance) were standardized across all subjects. Data points that fell more than three standard deviations away from the mean were considered outliers and removed from all further analyses.

A series of multiple linear regression and partial correlation analyses were conducted both within and across subjects (Hou et al., 2023), aiming to answer three questions:

- whether the stimulus parameters and electrode-retina interface properties could affect the perceived shape in single-electrode stimulation,
- whether the significant predictors were consistent across single-electrode stimulation and paired-electrode stimulation, and
- whether the shapes of phosphenes elicited by single-electrode stimulation would add up linearly in paired-electrode stimulation.

## 3. Results

### 3.1. Amplitude and frequency modulation affect single-point perception differently

Consistent with the literature on single-electrode phosphene drawings (Nanduri et al., 2008; Luo et al., 2016; Beyeler et al., 2019), phosphene shape greatly varied across subjects and electrodes, but was relatively consistent across trials of a single electrode. Single-electrode stimulation reliably elicited phosphenes in all three participants, who reported seeing a single phosphene on 86.7% of trials, two phosphenes on 13.1% of trials, and three or more phosphenes on the remaining trials.

**Figure 5:**
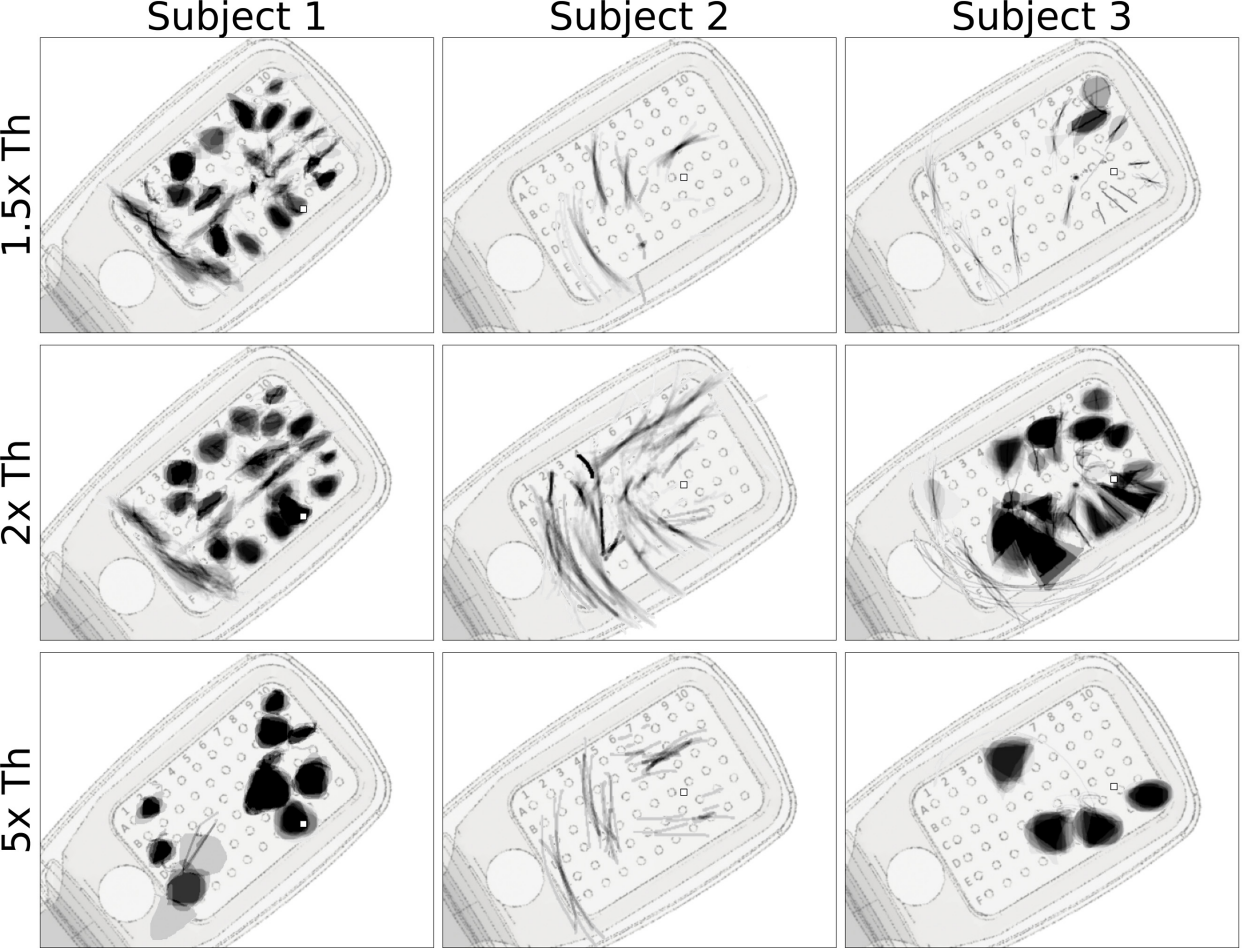
Single-electrode phosphene drawings as a function of stimulus amplitude (*rows*; expressed as multiples of the threshold current). Mean images were obtained by averaging drawings from individual trials aligned at their center of mass. Averaged drawings were then overlaid over the corresponding electrode in a schematic of each subject’s implant. Pulse train frequency was 20 Hz for all subjects. Squares (□) indicate the estimated location of the fovea.

Fig. 5 shows the mean images for each electrode, obtained by averaging the drawings for each electrode across trials obtained with a particular current amplitude (Fig. 5 *rows*; expressed as a multiple of the threshold current). Mean images were then centered over the corresponding electrode in a schematic of the subject’s implant to reveal the rich repertoire of elicited percepts across electrodes.

Whereas Subject 1 mostly drew blobs and wedges, which grew larger as the stimulus amplitude was increased, Subject 2 reported seeing exclusively lines and arcs, which got longer with increasing amplitude. The effect of amplitude on phosphene shape was most apparent for Subject 3, where phosphenes that appeared as lines and arcs near threshold turned into blobs and wedges as amplitude was increased.

**Figure 6:**
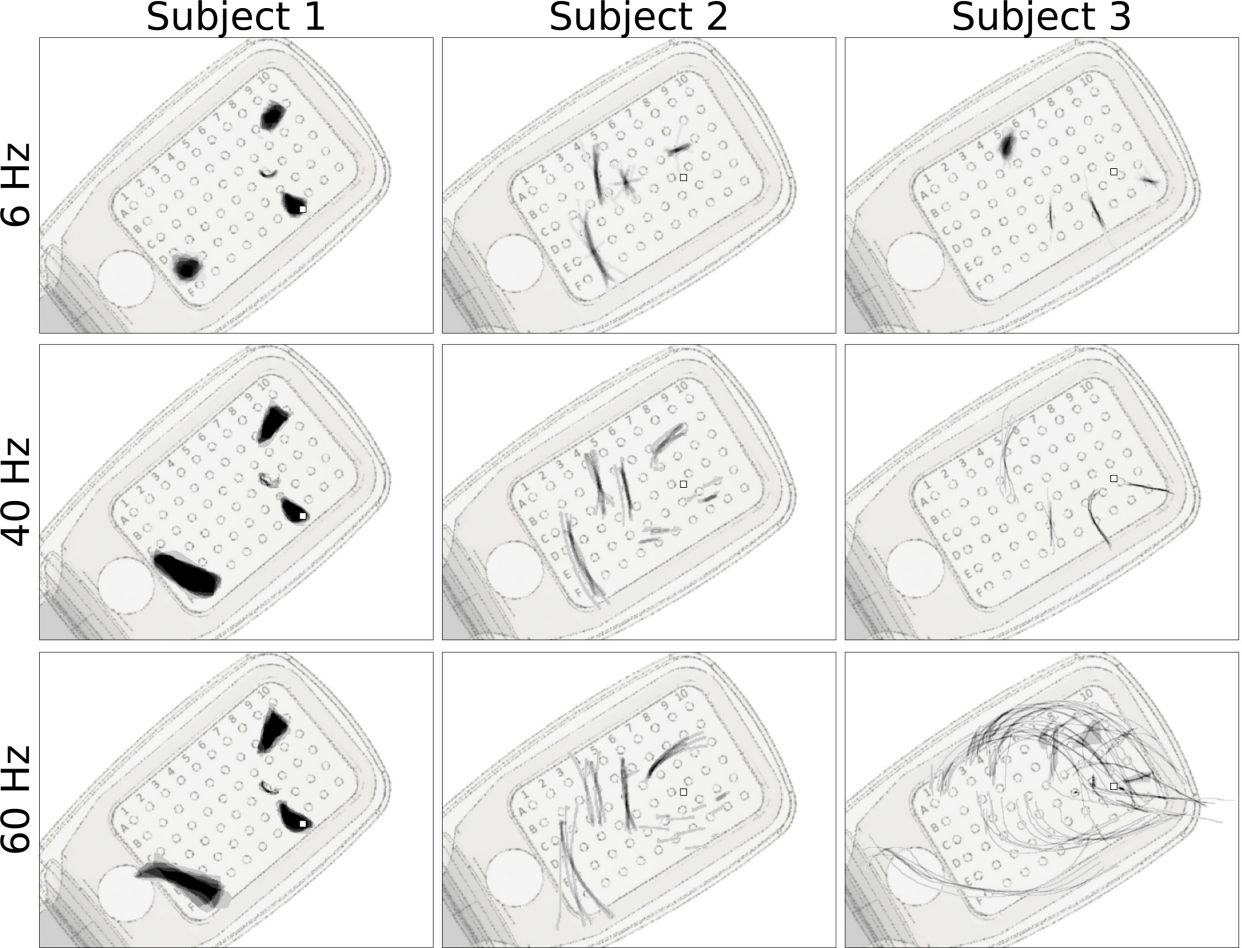
Single-electrode phosphene drawings as a function of pulse train frequency. Mean images were obtained by averaging drawings from individual trials aligned at their center of mass. These averaged drawings were then overlaid over the corresponding electrode in a schematic of each subject’s implant. Shown are only those electrodes for which drawings at all stimulus frequencies were available. Stimulus amplitude was 1.5 times threshold for Subjects 1 and 2, and 1.25 times threshold for Subject 3. Squares (□) indicate the estimated location of the fovea.

First reported by Nanduri et al. (2012), pulse frequency seemed to affect phosphene shape differently than amplitude (Fig. 6). Across all three subjects, phosphenes grew larger and/or more elongated as the pulse frequency increased. Whereas phosphenes that were located close to the center of vision (denoted by in □ Fig. 6) did not noticeably change in shape, more eccentric phosphenes turned from blobs at 6 Hz to rectangles at 60 Hz (Subject 1), or from short streaks at 6 Hz to orders-of-magnitude longer arcs at 60 Hz (Subject 2).

### 3.2. Predicting two-point perception from single-point perception

**Figure 7:**
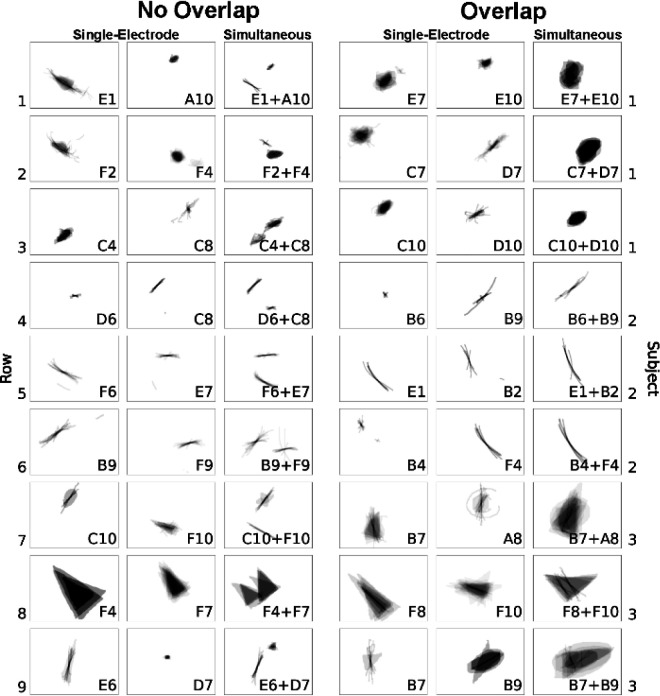
*Left*: Representative examples of single phosphenes combining linearly without overlap during paired-electrode stimulation. *Right*: Representative examples of phosphenes merging and overlapping during paired-electrode stimulation. Mean images were obtained by averaging drawings from individual trials aligned at their center of mass (see Appendix B5).

When two electrodes were stimulated simultaneously, participants reported seeing a single phosphene on 53.8% of trials, two phosphenes on 42.8% of trials, and three or more phosphenes on the remaining trials. Three or more phosphenes were generally encountered when single-electrode stimulation itself produced more than one phosphene. Representative examples of phosphene drawings for different electrode pairs are shown in Fig. 7, averaged across trials.

When paired-electrode stimulation produced two distinct phosphenes (Fig. 7, *left*), their shape resembled the linear summation of the phosphenes reported during single-electrode stimulation. For instance, as shown in Row 1 of the left panel in Fig. 7, Subject 1 perceived a long arc when electrode E1 was stimulated and an oval when electrode A10 was stimulated. When both E1 and A10 were stimulated concurrently, the resulting phosphene appeared as an arc alongside an oval. Similarly, in Row 9 of the left panel, Subject 3 perceived a tilted line for electrode E6 and a small triangle for electrode D7. Then during the simultaneous stimulation of electrodes E6 and D7, the resulting shape preserved the original form of the individual phosphene shapes.

When paired-electrode stimulation produced a single phosphene (Fig. 7, *right*), the phosphenes reported during single-electrode stimulation appeared to merge into a unified shape. For instance, as shown in Row 2 of the right panel in Fig. 7, Subject 1 perceived a blob for electrode C7 and a right-leaning straight line for electrode D7. When both C7 and D7 were stimulated simultaneously, the subject saw a larger blob tilted rightward. Similarly, in Row 6, electrode B4 elicited a small dot, and electrode F4 elicited a long arc; and simultaneous stimulation yielded an arc-shaped phosphene, appearing as a cohesive shape formed by connecting the two individual shapes.

Naturally, we asked to what extent the phosphene shape elicited by paired-electrode stimulation could be predicted by the phosphene shape elicited during single-electrode stimulation. To answer this question, we conducted a simple linear regression (Table 3), where each shape descriptor from a paired-electrode stimulation trial (e.g., the sum of phosphene areas when Electrodes A and B were simultaneously stimulated) was regressed on the same shape descriptor from a single-electrode stimulation trial (e.g., phosphene area elicited by Electrode A plus phosphene area elicited by Electrode B). In short, we found that each paired-electrode shape descriptor could be predicted by the sum of the two corresponding single-electrode shape descriptors (Table 3, p<.001). Across all subjects, shape descriptors tended to sum linearly, with the β values suggesting that phosphenes elicited by paired-electrode stimulation appeared larger than the average of their single-electrode counterparts, but smaller than their sum.

### 3.3. Factors affecting phosphene appearance during single-electrode stimulation

To more systematically investigate how different stimulus and anatomical parameters affect phosphene shape, we considered how the four shape descriptors (area, perimeter, major axis length, and minor axis length; see Methods, Section 2.4) could be predicted by different stimulus parameters (i.e., amplitude and frequency) and neuroanatomical parameters (i.e., electrode-retina distance and electrode-fovea distance). To address this, shape descriptor values were first averaged across trials and normalized per subject, then input to a multiple linear regression model (see Methods, Section 2.8).

The results are shown in Table 4 (for partial correlation plots, see Appendix F). Consistent with previous literature (Nanduri et al., 2012), we found that stimulus amplitude strongly affected phosphene area (β=.300,p<.001,r=.393), and to a lesser degree minor axis length (β=.207,p<.001,r=.341); suggesting that phosphenes get larger and blobbier with increasing amplitude. In contrast to Nanduri et al. (2012), we found that stimulus frequency also affected all four shape parameters (area: β=.120,p<.05,r=.129; perimeter: β=.429,p<.001,r=.510; major axis length: β=.596,p<.001,r=.515; minor axis length: β=.301,p<.001,r=.373).

In terms of neuroanatomical parameters, we considered an electrode’s distance to the retina (i.e., height) and distance to the fovea (i.e., retinal eccentricity). We found that electrode-*retina* distance had a significant effect on phosphene area (β=.154,p<.001,r=.274) and minor axis length (β=.103,p<.001,r=.228). However, due to data availability, this effect was solely based on the phosphene drawings of Subject 1, as the electrode-retina distance was zero for all electrodes of the other two subjects. Interestingly, we also found that electrode-*fovea* distance had a small but significant effect on all four shape parameters, making more peripheral phosphenes generally larger (area: β=.174,p<.001,r=.326; perimeter: β=.199,p<.001,r=.448; minor axis length: β=.131,p<.001,r=.305) and slightly longer (major axis length: β=.234,p<.001,r=.395; also see the bottom-right panel of Fig. 6).

As mentioned above, approximately 13% of single-electrode trials elicited multiple phosphenes. Multiple regression revealed that subjects were more likely to see multiple phosphenes as stimulus amplitude increased (β=.115,r=.309,p<.001; rightmost column of Table 4).

Phosphene orientation was previously shown to depend mostly on the retinal location of the stimulating electrode (Beyeler et al., 2019) and was thus excluded from the main analysis (the interested reader is referred to Appendix E). The per-subject linear regression analyses are reported in Appendix D. Due to the limited sample size within each subject, readers should use these tables with caution.

### 3.4. Factors affecting phosphene appearance during paired-electrode stimulation

We wondered whether these stimulus and neuroanatomical parameters could also explain the shape of phosphenes elicited by paired-electrode stimulation. As subjects would frequently draw multiple phosphenes during paired-electrode stimulation (Fig. 7), we extracted each shape descriptor for each individual phosphene. Then, we summed all phosphenes’ corresponding shape descriptor within each drawing in order to account for the variable number of perceived phosphenes. Finally, we averaged each shape descriptor of each drawing across trials (see Methods, Section 2.8).

**Figure 8:**
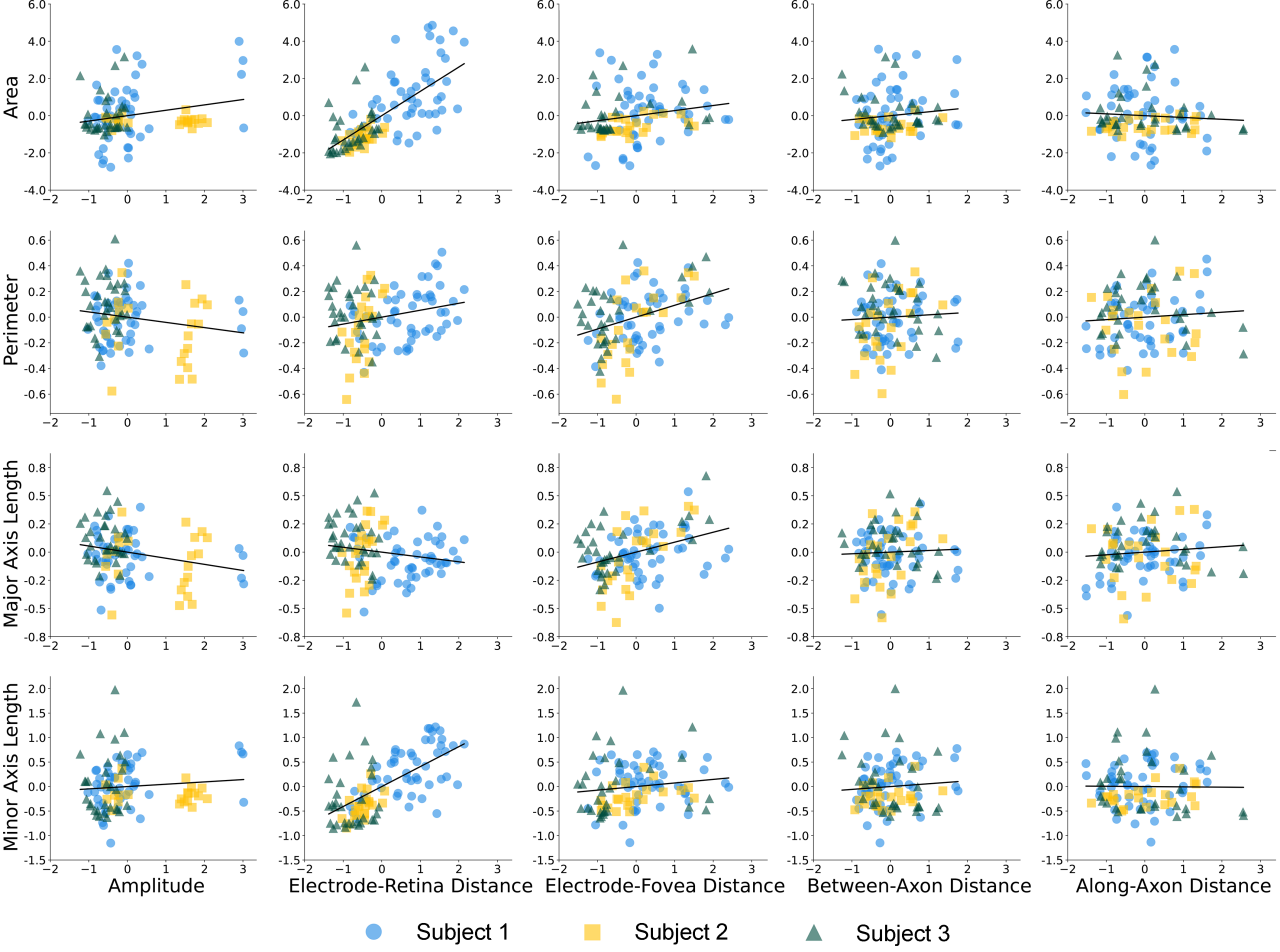
Partial correlation plots of normalized phosphene shape elicited by paired-electrode stimulation, correlated with standardized stimulus amplitude and neuroanatomical parameters (arbitrary units).

The results are shown in Table 5 and Fig. 8. Similar to the single-phosphene drawings, the average of stimulus amplitudes significantly increased the area (β=.289,p<.05,r=.224) and reduced major axis length (β=−.0542,p<.05,r=−.246). All paired-electrode drawings were collected at 20 Hz, thus frequency could unfortunately not be included in the regression. The average of two stimulating electrodes’ electrode-*retina* distances strongly increased phosphene area (β=1.310,p<.001,r=.701), perimeter (β=.0540,p<.05,r=.228) and minor axis length (β=.407,p<.001,r=.621), thus making phosphenes larger and rounder. Moreover, the average of two electrode-*fovea* distances in an electrode pair was also a strong predictor of paired-electrode phosphene shape, leading to an increase in phosphene area (β=.273,p<.05,r=.200), perimeter (β=.0924,p<.001,r=.369) and major axis length (β=.0882,p<.001,r=.362), but not minor axis length (p>.05). In addition, subjects were more likely to report seeing two phosphenes with shorter electrode-fovea distances (β=−.133,p<.01,r=−.294).

In terms of the inter-electrode distance, Yücel et al. (2022) previously demonstrated that the probability of perceiving two distinct phosphenes increases with inter-electrode distance. However, the axon map model (Beyeler et al., 2019) makes a more nuanced prediction: subjects should be more likely to see two distinct phosphenes as the distance between two nerve fiber bundles increases (“between-axon” distance; as opposed to distance on the retinal surface). Under this model, paired-electrode stimulation with a short between-axon distance should activate the same nerve fiber bundles and thus lead to a single phosphene, even though the two electrodes may be far apart on the retina.

To test this hypothesis, we split retinal distance into two, almost orthogonal components (see Methods, Section 2.5): “between-axon” distance, which spreads the current radially from the more nasal electrode until it reaches the more temporal electrode’s closest axon, and “along-axon” distance, which walks along the axon from that point until it reaches the more temporal electrode (see Methods). This works even for pairs on opposite sides of the raphe (see Fig. 3). During the preliminary stage of this study, we experimented with a number of similar formulations of splitting these two components, and all of them gave similar results.

Consistent with the axon map model (Beyeler et al., 2019), we found that between-axon distance was a significant predictor of the number of perceived phosphenes (β=.162,p<.05,r=.257; Table 5), but not along-axon distance (p>.05). Interestingly, neither the between-axon nor the along-axon distance significantly affected the phosphene shape (p>.05).

## 4. Discussion

In this study, we set out to investigate the relationship between single-point and two-point perception of Argus II users. Our results suggest that two-point perception can be predicted by the linear summation of single-point perception, supporting the notion of independent stimulation channels. We also found that the number of perceived phosphenes increased with the between-axon distance of two stimulating electrodes, but not the along-axon distance, thus providing further evidence in support of the axon map model for epiretinal stimulation (Rizzo et al., 2003; Nanduri, 2011; Beyeler et al., 2019).

These findings contribute to the growing literature on phosphene perception and have important implications for the design of future retinal prostheses.

### 4.1. Phosphene shape is well predicted by stimulus and neuroanatomical parameters

Although a link between electrode-retina distance and perceptual thresholds has been well established in the literature (Mahadevappa et al., 2005; de Balthasar et al., 2008; Ahuja et al., 2013; Pogoncheff et al., 2023), research examining the effect of electrode-retina distance on the *shape* of elicited phosphenes has been limited.

We found that phosphenes tended to appear larger and rounder as stimulus amplitude (and to a lesser degree, electrode-retina distance) increased (Table 4). This finding is consistent with previous considerations about current spread in the retina (de Balthasar et al., 2008; Granley and Beyeler, 2021; Yücel et al., 2022): as electrode-retina distance increases, larger currents are needed to activate the target neurons, which in turn activate larger areas of the retinal tissue.

In contrast to Nanduri et al. (2012), we found that stimulus frequency also affected phosphene size (Table 4 and Fig. 6). This discrepancy can potentially be explained by the fact that their 2012 analysis was limited to nine different electrodes from a single Argus I patient, whereas the current study sampled phosphenes from 90 different electrodes across three Argus II patients. Moreover, our results agree with data from suprachoroidal prostheses, where phosphenes tend to appear fuller (i.e., thicker or rounder) as the stimulation rate increases (Sinclair et al., 2016).

In addition, we found that electrode-fovea distance (i.e., retinal eccentricity) increased all four considered shape descriptors (area, perimeter, major/minor axis length; Table 4). This may simply be a consequence of ganglion cell receptive fields increasing with eccentricity (Curcio and Allen, 1990), which agrees with psychophysical (Freeman and Simoncelli, 2011; Stingl et al., 2013) and computational considerations (Song et al., 2022), but is an as-of-yet unpublished finding about the appearance of phosphenes elicited by retinal implants. Indeed, most phosphene models assume a constant scaling factor between retinal and visual field coordinates (Horsager et al., 2009; Nanduri, 2011; Beyeler et al., 2019).

### 4.2. Two-point perception is the linear sum of single-point perception

This study demonstrates that the phosphene shape in paired-electrode stimulation can be predicted by the stimulus and neuroanatomical parameters that describe the single-electrode phosphene shape (Table 5). However, there are some disparities between predictors for single-electrode and paired-electrode phosphenes. For example, stimulus amplitude significantly predicted the minor axis length in single-electrode stimulation, but not in paired-electrode stimulation. Electrode-fovea distance accounted for all four shape descriptors in single-electrode stimulation, but only significantly predicted three descriptors during paired-electrode stimulation. Such disparities indicate the presence of more complex mechanisms mediating the effect of each factor on phosphene shape during paired-electrode stimulation, which cannot be captured by a linear model.

On the perceptual level, we found that the shape of phosphenes elicited by paired-electrode stimulation was well predicted by the linear summation of the shape of their corresponding single-electrode phosphenes (Table 3), supporting the notion of independent channels for phosphene perception. Specifically, β values in Table 3 suggested that phosphenes elicited by paired-electrode stimulation were smaller than the sum of their single-electrode counterparts. These findings are partially consistent with Christie et al. (2022), who showed that the phosphene elicited by electrode “quads” was similar to phosphenes elicited by individual electrodes that belonged to the quad, with Wilke et al. (2011a), who showed that single-electrode phosphenes consisting of round dots and lines added up to more complicated patterns when stimulated simultaneously, and with Barry et al. (2020), who reported that multi-electrode percepts in the Orion cortical implant were perceived to be smaller and simpler than the predicted combination of single-electrode phosphene shapes.

The observed linear summation of single-electrode phosphenes provides a valuable direction for future computational model development, particularly beneficial when predicting phosphenes in multi-electrode stimulation scenarios (Zrenner et al., 2010; Shivdasani et al., 2017). However, it should be noted that multiple phosphene patterns may not automatically group into perceptually intelligible objects (Stingl et al., 2015; Shivdasani et al., 2017; Christie et al., 2022). This “binding problem” (Roelfsema, 2023) also extends to cortical implants. Although a recent study with intracortical electrodes (Chen et al., 2020) showed that macaques could successfully identify the intended shape of a patterned electrical stimulus, human subjects implanted with the same technology could not always do that (Fernández et al., 2021). In contrast, human subjects implanted with cortical surface electrodes required a dynamic stimulation strategy to allow for perceptual grouping (Beauchamp et al., 2020).

### 4.3. The number of perceived phosphenes depends on the axonal distance in paired-electrode stimulation

We found that the number of perceived phosphenes tended to increase as the between-axon distance of two stimulating electrodes increased (Table 5). While it is not surprising that two electrodes separated by a large retinal distance might produce two distinct phosphenes (Yücel et al., 2022), here we were able to split retinal distance into two (nearly orthogonal) components: between-axon distance, which measures how far the electric current must spread *away from* an axon bundle until it reaches another electrode, and along-axon distance, which measures how far the electric current must spread *along* an axon bundle until it reaches another electrode. Our results provide the first computational evidence that paired-electrode stimulation is more likely to elicit two distinct phosphenes as the distance between their underlying axon bundles increases. This provides further evidence in support of the axon map model for epiretinal stimulation (Rizzo et al., 2003; Nanduri, 2011; Beyeler et al., 2019).

### 4.4. Limitations and future work

Despite the ability of our model to highlight important factors that guide the appearance of phosphenes elicited by retinal implants, it is important to note that our linear regression analyses cannot identify nonlinear predictors of phosphene shape. Future studies could thus focus on nonlinear (but still explainable) machine learning models (Pogoncheff et al., 2023). In addition, due to data availability, our analyses are currently limited to single- and paired-electrode stimulation. However, to achieve pattern vision, it will be important to stimulate more than two electrodes at a time. Therefore, future studies should investigate whether this linear summation can be extended to more than two electrodes.

## Figures and Tables

**Table 1:** Subject details: clinical implant site, sex (M: male, F: female), preoperative visual acuity (VA) categorized as either bare light perception (BLP) or no light perception (NLP), the age range at implantation, and the number of years that subjects had been blind prior to implant surgery (self-reported). Years blind for Subject 1 was unknown due to gradual loss of vision.

Subject ID	Sex	Pre-op VA	Age range at surgery	Years blind

1	M	NLP	61–70	?
2	F	NLP	41–50	11–20
3	M	BLP	41–50	21–30

**Table 2:** Each subject’s number of sampled electrodes, electrode-fovea distance (mean ± SEM), and electrode-retina distance (mean ± SEM).

Subject	Number of included electrodes	Electrode-fovea distance (μm)	Electrode-retina distance (μm)

1	30	2561.0 ± 217.5	150.9 ± 25.5
2	30	2136.2 ± 173.1	0.0
3	30	2168.8 ± 227.4	0.0

**Table 3:** Linear regression and correlation analysis of paired-electrode shape descriptors predicted by the corresponding single-electrode shape descriptor (106 mean drawings).

Single-electrode shape	Paired-electrode shape									
	Area		Perimeter		Major axis length		Minor axis length		Num phosphenes	
				
	β	r	β	r	β	r	β	r	β	r

Area	**.630** ***	**.579**	-	-	-	-	-	-	-	-
Perimeter	-	-	**.611** ***	**.733**	-	-	-	-	-	-
Major axis length	-	-	-	-	**.587** ***	**.722**	-	-	-	-
Minor axis length	-	-	-	-	-	-	**.675** ***	**.753**	-	-
Num phosphenes	-	-	-	-	-	-	-	-	**.647** ***	**.288**

β: standardized regression coefficient. r: partial correlation coefficient.

***: *p* < .001.

Significant effects are marked in bold. Intercepts were not included in the analysis, because if the value of a predictor (sum of the single-electrode phosphene shapes) was zero, the corresponding value of the dependent variable (the paired-electrode phosphene shape) should also be zero.

**Table 4:** Multiple regression and partial correlation analysis of single-electrode phosphene shape and numbers (255 mean drawings) predicted by amplitude, frequency, electrode-retina distance, and electrode-fovea distance. The variance inflation factor of all predictors was smaller than 1.5, suggesting minimal multicollinearity.

	Area		Perimeter		Major axis length		Minor axis length		Number of phosphenes	
					
	β	r	β	r	β	r	β	r	β	r

Amplitude	**.300** ***	**.393**	.0510	.0919	−.0404	−.0534	**.207** ***	**.341**	**.115** ***	**.309**
Frequency	**.120** *	**.129**	**.429** ***	**.510**	**.596** ***	**.515**	**.301** ***	**.373**	.0361	.0774
Electrode-retina distance	**.154** ***	**.274**	.0185	.0432	−.0393	−.0673	**.103** ***	**.228**	−.0156	−.0568
Electrode-fovea distance	**.174** ***	**.326**	**.199** ***	**.448**	**.234** ***	**.395**	**.131** ***	**.305**	−.0158	−.0620

*: *p* < .05

**: *p* < .01

***: *p* < .001.

Significant effects are marked in bold. Intercepts (not shown) were included in the analysis.

**Table 5:** Multiple linear regression and partial correlation analysis of paired-electrode phosphene shapes and numbers (103 mean drawings) predicted by amplitude, electrode-retina distance, electrode-fovea distance, between-axon distance, and along-axon distance. On trials that elicited multiple phosphenes, factors and shape descriptors were first extracted for each individual phosphene, before being averaged. The variance inflation factor of all predictors was smaller than 1.5, suggesting minimal multicollinearity.

	Area		Perimeter		Major axis length		Minor axis length		Number of phosphenes	
					
	β	r	β	r	β	r	β	r	β	r

Amplitude	**.289** *	**.224**	−.0406	−.182	**−.0542** *	**−.246**	.0475	.0969	−.0740	−.179
Electrode-retina distance	**1.310** ***	**.701**	**.0540** *	**.228**	−.0429	−.187	**.407** ***	**.621**	−.0713	−.164
Electrode-fovea distance	**.273** *	**.200**	**.0924** ***	**.369**	**.0882** ***	**.362**	.0731	.140	**−.133** **	**−.294**
Between-axon distance	.209	.110	.0182	.0556	.0146	.0457	.0580	.0792	**.162** *	**.257**
Along-axon distance	−.0975	−.0700	.0192	.0792	.0237	.100	−.00605	−.0112	−.00548	−.0122

*: *p* < .05

**: *p* < .01

***: *p* < .001.

Significant effects are marked in bold. Intercepts (not shown) were included in the analysis.

**Figure B1:**
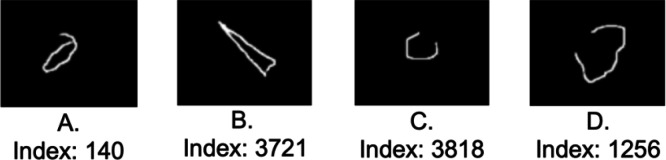
Examples of phosphene drawings with open contour lines.

**Figure B2:**
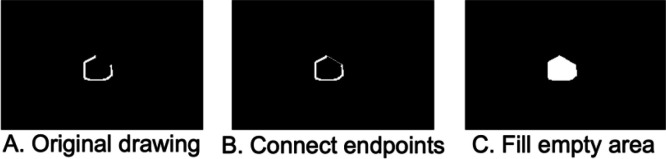
Procedure for fixing phosphene drawings with open contour lines.

**Figure B3:**
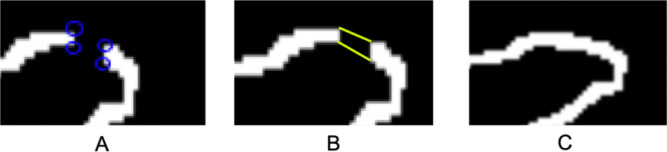
Procedure for fixing phosphene drawings with broken contour lines.

**Figure B4:**
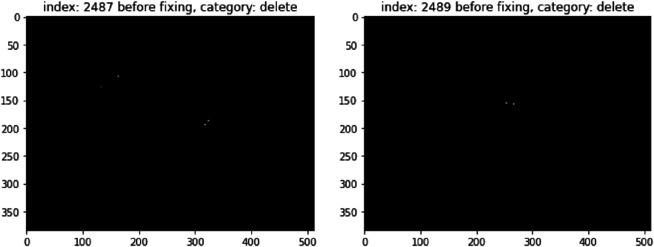
Example phosphene drawings with small specs (artifacts) that were removed from the dataset.

**Figure B5:**
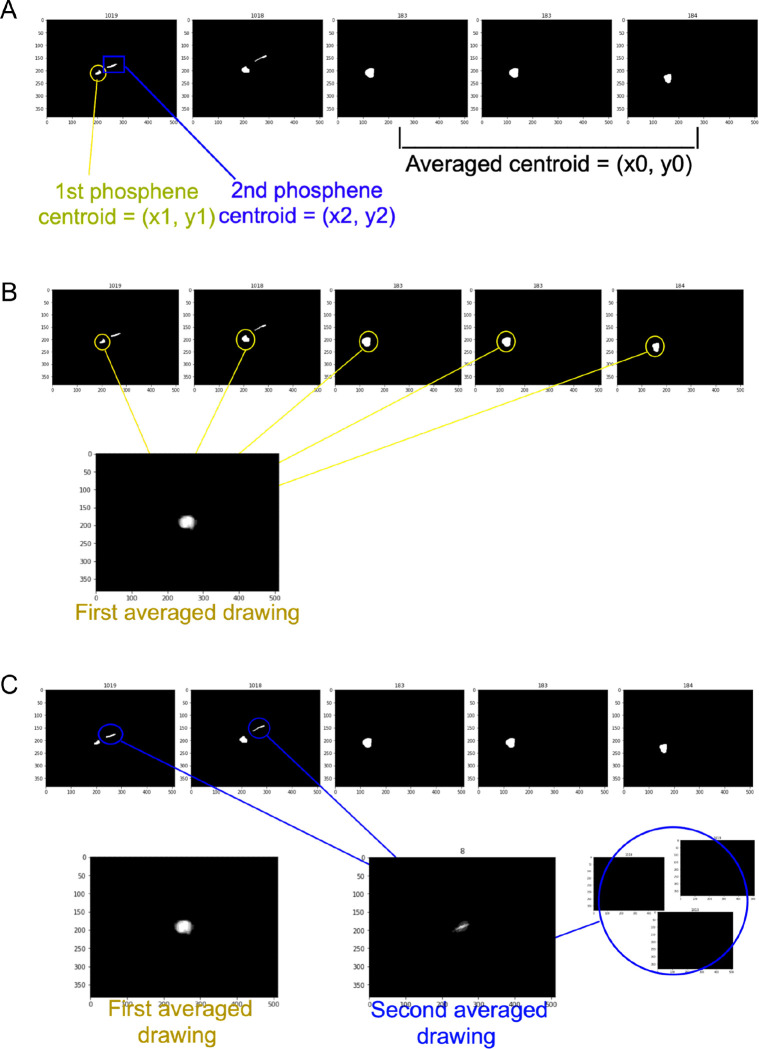
Procedure for stacking drawings with varying numbers of phosphenes.

**Figure C1:**
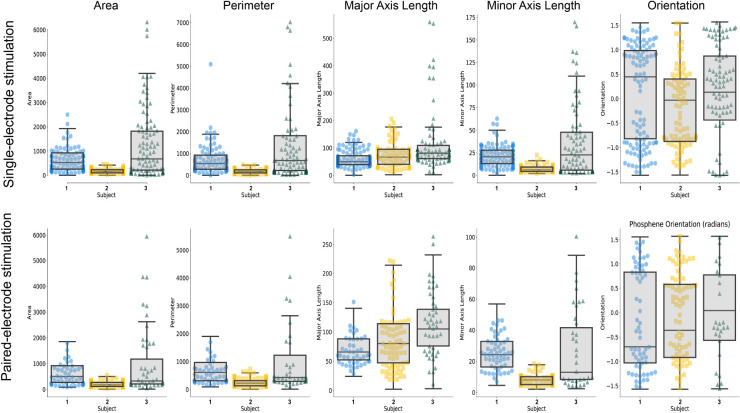
Boxplot of different phosphene shape properties for single-electrode stimulation (top row) and paired-electrode stimulation (bottom row).

**Figure F1:**
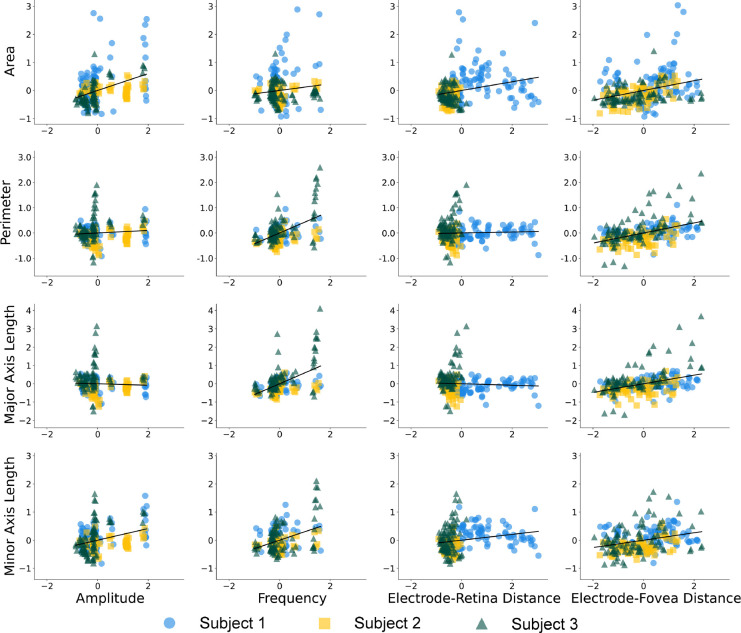
Partial correlation plots of area, perimeter, major axis length, or minor axis length correlated with amplitude, frequency, electrode-retina distance, and electrode-fovea distance across all subjects in single-electrode stimulation.

**Figure F2:**
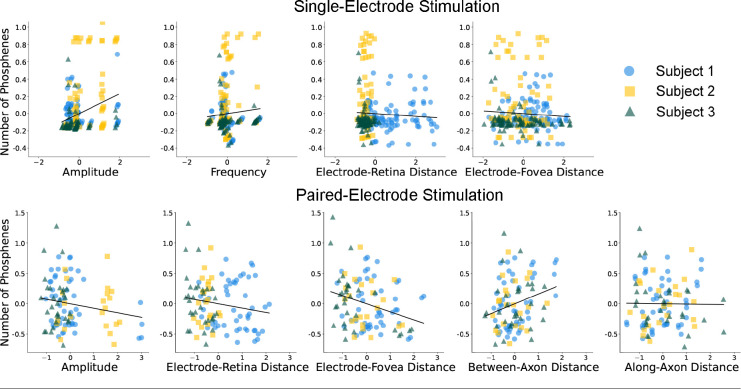
Partial correlation plots of the number of distinct phosphene regions correlated with stimulus parameters and electrode-retina properties across all subjects in single-electrode (first row) and paired-electrode (second row) stimulation.

## Data Availability

The code to produce the figures and tables is publicly available at https://github.com/bionicvisionlab/2023-ArgusPairs. Data will be made publicly available via Open Science Framework upon acceptance of this article.

## Appendix A.

### Perceptual threshold measurements

Custom software was used to measure the perceptual thresholds on each electrode using a yes-no procedure that was a hybrid between an adaptive staircase and a method of constant stimuli (de Balthasar et al., 2008). Stimuli were charge-balanced, 0.45 ms per phase, cathodic-first, biphasic 20 Hz pulse trains, 250 ms in duration.

The experiment consisted of five sessions, and each electrode was tested 12 times in each session, in random order. 32 catch trials were also interspersed randomly over five sessions to minimize the false alarm rate. Upon stimulation, subjects had to report whether they were able to see any phosphenes (detection task). Stimulus amplitudes (for stimulus present trials) for the first block were predetermined (method of constant stimuli). After the first block, a maximum likelihood algorithm fit of a Weibull function to the current data determined the range of the next block of stimulation amplitude values for each electrode. After each block, a confidence interval was acquired for each electrode using a Monte-Carlo simulation based on responses to the previous trials. If the confidence interval for an electrode fell below a pre-set level, trials for that electrode were no longer presented, but trials on the other electrodes continued through a maximum of five blocks.

Results were deemed unreliable if the false alarm rate, determined by the percentage that the subject saw a stimulus during catch trials, was greater than 20%. Data from runs with higher false alarm rates than 20% were removed from the analysis and the runs were repeated.

## Appendix B.

### Data cleaning

#### Appendix B.1. Phosphene drawings with open contour lines

It was sometimes challenging for our subjects (who were completely blind) to draw fully closed circles, triangles, or wedges. Although a common strategy is to place one finger at the starting location while the other finger traces out the shape (thus simplifying the process of “returning home” and closing the contour), some drawings ended up with open contour lines (Fig. B1). These drawings were identified as follows:

- The drawing was either a hollow circle or triangle and either had a small gap between two endpoints (Panels A and B) or a line that resembled the shape of a circle or triangle (Panels C and D).
- The majority of drawings from the same electrode showed similarly shaped phosphenes which were all filled.

Based on these criteria, we identified 21 (out of 4402) drawings that needed to be fixed. The data cleaning process involved three steps (Fig. B2):

- Identify the two endpoints of the drawing (Panel A).
- Connect the two endpoints with a 1px-thick line (Panel B).
- Fill the area with scipy.ndimage.binary_fill_holes() (Panel C).

#### Appendix B.2. Phosphene drawings with broken contour lines

Similarly, some phosphenes did have small gaps in otherwise smooth contour lines (most likely a tracking issue with the touchscreen). These small artifacts could potentially have grave effects on our phosphene shape analysis, as a gap in the contour line would accidentally double the number of reported phosphenes and halve their reported size.

Fortunately, we identified only twelve phosphene drawings with this issue. These drawings were identified by having a different number of phosphenes than drawings from other trials, caused by a small gap (on average approximately 10px wide; much smaller than the overall phosphene size). To fix them, we manually identified four endpoints of the broken contour line (Fig. B3, Panel A) and connected them (Panel B), then used scipy.ndimage.binary_fill_holes() to fill the area (Panel C).

#### Appendix B.3. Phosphene drawings with other artifacts

Nine phosphene drawings had other artifacts, such as tiny specs (less than 20 pixels in size) that were not part of any other discernible shape, and were subsequently removed (Fig. B4).

#### Appendix B.4. Stacking and averaging phosphene drawings

Cleaned phosphene drawings were grouped by subject, stimulating electrode(s), stimulus frequency(s), and stimulus amplitude(s); thus typically yielding a total of five drawings from five trials per group.

We used the measure module of scikit-image to automatically extract the number of phosphenes (number of connected regions) and their corresponding centroids. To draw average phosphenes for a particular electrode (e.g., Fig. 5), phosphenes were aligned by their centroid and averaged, then aligned with the electrode location of the implant schematic. To predict the shape parameters with multiple linear regression (e.g., Table 4), we first calculated all shape parameters for each individual phosphene, and then averaged the values for the shape parameters (as opposed to extracting the shape parameters from the averaged phosphene drawing).

If all five trial drawings showed exactly one phosphene, alignment and averaging were straightforward. Of all five trial drawings showed exactly two phosphenes, phosphenes were assigned to electrodes by clustering their centroid locations:

- In drawing of Trial 1, label the phosphene with the smaller centroid location as the “first” phosphene, and the other as the “second”.
- In drawing of Trial 2, extract the centroid location of the first phosphene and compare it to the centroid locations from Trial 1. Assign it to the “first” or “second” phosphene depending on which centroid is closest. Repeat for the second phosphene.
- Repeat the process until all trials are done.

If the number of phosphenes varied across drawings from different trials, a more sophisticated procedure was necessary:

- Find the average centroid location of all single-phosphene drawings (Fig. B5, Panel A). In the paired-phosphene drawings, identify the phosphene that is closest to the average centroid location (“first phosphene centroid”).
- Find all phosphenes belonging to that centroid and average them (Panel B, “first averaged drawing”).
- Find all other phosphenes that have not been processed yet and average those (Panel C, “second averaged drawing”).

Note that all average drawings resulting from this process were manually examined to ensure the integrity of the process.

## Appendix C.

### Distribution of phosphene shape descriptors

Fig. C1 shows the distribution of shape descriptors.

Phosphene drawings were more consistent within than across subjects (for details, see Nanduri (2011); Beyeler et al. (2019)). In single-electrode stimulation, 1 tended to draw simple dots, oval and elongated lines varying in length and thickness (Fig. 5; *left column*). However, round, or oval shapes never appeared in Subject 2’s drawings as all phosphenes were curved or straight lines (Fig. 5; *center column*), leading Subject 2’s average phosphene areas, minor axis lengths, and perimeters to be much smaller than those of the other two subjects. This was also evident in the boxplots of each subject’s phosphene shapes (Fig. C1; *top row*), because Subject 2’s median area, minor axis length, and perimeter were even smaller than those of other subjects’ 25th quantile. The drawings of Subject 3 (that included curved lines, ovals, and triangles) varied dramatically in shape across different electrodes. Similar tendencies were observed in paired-electrode stimulation (Fig. C1, *bottom row*).

## Appendix D.

### Per-Subject analysis

We systematically analyzed the phosphene shape and numbers in single- and paired-electrode stimulation for each subject. Due to the small sample size, readers should view tables and use the information with caution.

**Table D1:** Multiple regression and partial correlation analysis of single-electrode phosphene shape and numbers predicted by amplitude, frequency, electrode-retina distance, and electrodefovea distance. The variance inflation factor of all predictors was smaller than 2.

		Area		Perimeter		Major axis length		Minor axis length		Number of phosphenes	
					
		β	r	β	r	β	r	β	r	β	r

Subject 1 (N=102)	Amplitude	**.406** ***	**.469**	**.0667** *	**.234**	−.0159	−.0499	**.266** ***	**.564**	.00566	.0296
	Frequency	**.468** ***	**.345**	**.117** *	**.245**	**.114** *	**.210**	.104	.158	−.0461	−.143
	Electrode-retina distance	**−.201** *	**−.253**	−.0261	−.0931	−.0101	−.0314	**−.114** **	**−.279**	**.0585** **	**.291**
	Electrode-fovea distance	−.0646	−.0830	**.0848** **	**.288**	**.125** ***	**.360**	−.0751	−.186	.0355	.180
Subject 2 (N=69)	Amplitude	**.0567** *	**.266**	**.113** ***	**.457**	**.121** ***	**.477**	**.0707** **	**.339**	**.215** ***	**.519**
	Frequency	.0927	.197	**.163** **	**.314**	**.180** **	**.339**	**.126** *	**.277**	**.320** **	**.374**
	Electrode-retina distance	–	–	–	–	–	–	–	–	–	–
	Electrode-fovea distance	**.166** ***	**.635**	**.162** ***	**.602**	**.170** ***	**.614**	**.103** ***	**.473**	−.0135	−.0388
Subject 3 (N=91)	Amplitude	**.490** ***	**.725**	**.219** *	**.268**	.0459	.0437	**.493** ***	**.522**	.00787	.0393
	Frequency	.0488	.117	**.673** ***	**.693**	**.930** ***	**.706**	**.506** ***	**.576**	−.0149	−.0833
	Electrode-retina distance	–	–	–	–	–	–	–	–	–	–
	Electrode-fovea distance	**.0651** *	**.244**	**.346** ***	**.619**	**.505** ***	**.654**	**.179** ***	**.369**	**−.0269** *	**−.234**

β: standardized regression coefficient. r: partial correlation coefficient. N: number of mean drawings. –: data not available.

*: *p* < .05

**: *p* < .01

***: *p* < .001.

Significant effects are marked in bold. Intercepts (not shown) were included in the analysis.

**Table D2:** Multiple linear regression and partial correlation analysis of paired-electrode phosphene shape and numbers predicted by amplitude, electrode-retina distance, electrodefovea distance, between-axon distance, and along-axon distance. For trials that elicited multiple phosphenes, factors and shape descriptors were first extracted for each individual phosphene, before being averaged. The variance inflation factor of all predictors was smaller than 3.

		Area		Perimeter		Major axis length		Minor axis length		Number of phosphenes	
					
		β	r	β	r	β	r	β	r	β	r

Subject 1 (N=46)	Amplitude	1.300	.292	.158	.280	.0590	.107	**.439** *	**.353**	−.150	−.126
	Electrode-retina distance	**.866** *	**.363**	**.101** *	**.337**	.0365	.126	**.267** **	**.403**	.0881	.142
	Electrode-fovea distance	−.0843	−.0384	**.098** *	**.331**	**.118** *	**.385**	−.0700	−.116	.0338	.0556
	Between-axon distance	.474	.208	.0285	.0999	−.00687	−.0238	**.203** *	**.317**	**.327** **	**.469**
	Along-axon distance	−.0640	−.0451	**.0653** *	**.340**	.0554	.290	.0568	.145	.108	.266
Subject 2 (N=22)	Amplitude	.0225	.109	.0204	.100	.0224	.110	.00594	.0355	.0198	.0504
	Electrode-retina distance	–	–	–	–	–	–	–	–	–	–
	Electrode-fovea distance	**.217** ***	**.716**	**.217** ***	**.722**	**.222** ***	**.729**	**.103** *	**.512**	−.059	−.145
	Between-axon distance	**.192** *	**.566**	**.192** *	**.574**	**.193** *	**.574**	**.179** **	**.619**	.150	.270
	Along-axon distance	.113	.374	.104	.354	.100	.342	**.167** **	**.593**	.116	.213
Subject 3 (N=31)	Amplitude	**.392** *	**.409**	.0647	.353	−.00471	−.0264	**.291** **	**.482**	.0841	.220
	Electrode-retina distance	–	–	–	–	–	–	–	–	–	–
	Electrode-fovea distance	.110	.0948	**.110** *	**.437**	**.142** **	**.517**	−.00170	−.00244	**−.224** *	**−.415**
	Between-axon distance	**−.761** *	**−.416**	−.0860	−.255	−.00197	−.00581	−.354	−.332	.170	.233
	Along-axon distance	.177	.126	−.0120	−.0439	−.0257	−.0899	.0173	.0206	−.187	−.299

–: data not available.

*: *p* < .05

**: *p* < .01

***: *p* < .001.

Significant effects are marked in bold. Intercepts (not shown) were included in the analysis.

**Table D3:** Linear regression and correlation analysis of paired-electrode stimulated shape predicted by electrode-electrode stimulated shape.

	Area		Perimeter		Major axis length		Minor axis length		Number of phosphenes	
					
	β	r	β	r	β	r	β	r	β	r

Subject 1 (N=52)	**.683** ***	**.389**	**.597** ***	**.555**	**.553** ***	**.720**	**.709** ***	**.554**	**0.627** ***	**0.314**
Subject 2 (N=22)	**.706** ***	**.905**	**.708** ***	**.903**	**.708** ***	**.903**	**.639** ***	**.646**	**0.568** ***	**0.389**
Subject 3 (N=33)	**.499** ***	**.652**	**.535** ***	**.673**	**.506** ***	**.592**	**.515** ***	**.589**	**0.755** ***	**0.492**

*: *p* < .05

**: *p* < .01

***: *p* < .001.

Significant effects are marked in bold. Intercepts were not included in the analysis.

## Appendix E.

### Orientation analysis

Phosphene orientation was also calculated from the covariance matrix of the phosphene drawing:

(E.1)
$$cov[I(x,\;y)]=\left[\begin{array}{ll} \mu_{20}^{\prime } & \mu_{11}^{\prime }\\ \mu_{11}^{\prime } & \mu_{02}^{\prime } \end{array}\right],$$

where ${mu}_{20}^{\prime }=M_{20}/M_{00}-{\overset{‾}{x}}^{2}$, $\mu_{11}^{\prime }=M_{11}/M_{00}-\overset{‾}{x}\overset{‾}{y}$, and $\mu_{02}^{\prime }=M_{02}/M_{00}-{\overset{‾}{y}}^{2}$. The eigenvectors of this matrix corresponded to the major and minor axes of the image intensity. Phosphene orientation could be extracted from the angle of the eigenvector associated with the largest eigenvalue towards the axis closest to this eigenvector:

(E.2)
$$\theta =\frac{1}{2}\arctan\left(\frac{2\mu_{11}^{\prime }}{\mu_{20}^{\prime }-\mu_{02}^{\prime }}\right),$$

which was valid as long as $\mu_{20}^{\prime }\neq \mu_{02}^{\prime }$, with θ∈[−π/2,π/2]. To avoid division by zero, we manually assigned an angle of θ=0 whenever $\mu_{20}^{\prime }$ was equal to $\mu_{02}^{\prime }$.

Consistent with Beyeler et al. (2019), we found a significant correlation between the orientation of the nerve fiber bundle closest to the stimulating electrode and the orientation of the perceived phosphene (Table E1). This was true for both single-electrode and paired-electrode stimulation experiments (p<.01; first two modules of Table E1). Moreover, the average of orientations in a paired-electrode stimulus could be predicted by the average orientations of the individual phosphenes measured during single-electrode stimulation (p<.001; last module of Table E1), suggesting that the orientation of individual phosphenes did not change much during simultaneous stimulation.

**Table E1:** Phosphene numbers predicted by different stimuli and electrode-retina interface properties in single-electrode drawings and pairedelectrode drawings, and phosphene numbers in paired-electrode drawings predicted by phosphene numbers in single-electrode drawings. The variance inflation factor of all predictors was smaller than 3.

		Single-electrode elicited phosphene ATL			Paired-electrode elicited phosphene ATL			Paired-electrode elicited phosphene ATL	
			
		β	r		β	r		β	r

Subject 1	Amplitude	**.283** *	**.231**	Amplitude	.321	.114	Single-electrode elicited phosphene axonal tangential line	**.701** ***	**.717**
	Frequency	−.172	−.0863	Frequency	-	-			
	Electrode-retina distance	.221	.181	Electrode-retina distance	.391	.272			
	Electrode-fovea distance	.185	.142	Electrode-fovea distance	.386	.251			
	Axonal tangential line	**.392** ***	**.364**	Axonal tangential line	**.408** **	**.415**			
		N=102			N=48			N=52	
Subject 2	Amplitude	−.178	−.227	Amplitude	.102	.109	Single-electrode elicited phosphene axonal tangential line	**.604** **	**.652**
	Frequency	−.390	−.221	Frequency	-	-			
	Electrode-retina distance	-	-	Electrode-retina distance	-	-			
	Electrode-fovea distance	−.147	−.154	Electrode-fovea distance	−.206	−.224			
	Axonal tangential line	**.636** ***	**.563**	Axonal tangential line	**.704** **	**.655**			
		N=69			N=22			N=22	
Subject 3	Amplitude	.184	.101	Amplitude	−.156	−.158	Single-electrode elicited phosphene axonal tangential line	**.653** ***	**.662**
	Frequency	−.0841	−.0518	Frequency	-	-			
	Electrode-retina distance	-	-	Electrode-retina distance	-	-			
	Electrode-fovea distance	−.0300	−.0297	Electrode-fovea distance	−.369	−.356			
	Axonal tangential line	**.471** ***	**.419**	Axonal tangential line	**.535** **	**.509**			
		N=91			N=32			N=32	
All Subjects	Amplitude	.0539	.0396	Amplitude	−.0876	−.0869	Single-electrode elicited phosphene axonal tangential line	**.675** ***	**.685**
	Frequency	−.131	−.0732	Frequency	-	-			
	Electrode-retina distance	−.0140	−.0126	Electrode-retina distance	−.0185	−.0164			
	Electrode-fovea distance	−.0387	−.0390	Electrode-fovea distance	−.112	−.111			
	Axonal tangential line	**.503*****	**.436**	Axonal tangential line	**.502** ***	**.469**			
		N=255			N=89			N=106	

*ATL*: Axonal Tangential Line.

*: *p* < .05

**: *p* < .01

***: *p* < .001.

Significant effects are marked in bold. Intercepts were not included in the analysis.
